# Radio-immune response modelling for spatially fractionated radiotherapy

**DOI:** 10.1088/1361-6560/ace819

**Published:** 2023-08-07

**Authors:** Young-Bin Cho, Nara Yoon, John H Suh, Jacob G Scott

**Affiliations:** 1 Department of Radiation Oncology, Taussig Cancer Institute, Cleveland Clinic, Cleveland, United States of America; 2 Department of Radiation Oncology, Cleveland Clinic Lerner College of Medicine of Case Western Reserve University, Cleveland, United States of America; 3 Department of Biomedical Engineering, Cleveland Clinic Lerner College of Medicine of Case Western Reserve University, Cleveland, United States of America; 4 Departmentof Mathematics and Computer Science, Adelphi University, New York, United States of America; 5 Department of Translational Hematology and Oncology Research, Cleveland Clinic Lerner College of Medicine of Case Western Reserve University, Cleveland, United States of America; 6 Department of Physics, Case Western Reserve University, Cleveland, United States of America

**Keywords:** radio immune response modelling, spatially fractionated RT, immune trap, immune escape, immune bifurcation, terminal tumor volume

## Abstract

*Objective.* Radiation-induced cell death is a complex process influenced by physical, chemical and biological phenomena. Although consensus on the nature and the mechanism of the bystander effect were not yet made, the immune process presumably plays an important role in many aspects of the radiotherapy including the bystander effect. A mathematical model of immune response during and after radiation therapy is presented. *Approach.* Immune response of host body and immune suppression of tumor cells are modelled with four compartments in this study; viable tumor cells, T cell lymphocytes, immune triggering cells, and doomed cells. The growth of tumor was analyzed in two distinctive modes of tumor status (immune limited and immune escape) and its bifurcation condition. *Main results.* Tumors in the immune limited mode can grow only up to a finite size, named as terminal tumor volume analytically calculated from the model. The dynamics of the tumor growth in the immune escape mode is much more complex than the tumors in the immune limited mode especially when the status of tumor is close to the bifurcation condition. Radiation can kill tumor cells not only by radiation damage but also by boosting immune reaction. *Significance.* The model demonstrated that the highly heterogeneous dose distribution in spatially fractionated radiotherapy (SFRT) can make a drastic difference in tumor cell killing compared to the homogeneous dose distribution. SFRT cannot only enhance but also moderate the cell killing depending on the immune response triggered by many factors such as dose prescription parameters, tumor volume at the time of treatment and tumor characteristics. The model was applied to the lifted data of 67NR tumors on mice and a sarcoma patient treated multiple times over 1200 days for the treatment of tumor recurrence as a demonstration.

## 1. Introduction

Cells irradiated with sufficient energy of photons or charged particles are lethally damaged through physical, chemical and biological processes spanning time scales from pico-seconds to days (Tubiana *et al* 1986, Zakaria *et al* 2020). Irreparable cells from the damage will die and be removed from tissues with some time delay (Lim *et al* 2008, Zhong and Chetty 2014). Radiotherapy of cancers has been evolved in a way to maximize the radiation damage to tumors respecting surrounding normal tissue tolerances. Considering tumor control probability sensitive to the minimum dose, extra efforts have been paid for the development of radiotherapy plans with homogeneous dose distribution conforming to targets. In recent decades, a new treatment delivery technique, FLASH (Zakaria *et al* 2020, Chow *et al* 2021, Lv *et al* 2022), using extremely high dose rate has reported a larger therapeutic gain than a standard radiotherapy due to the increased normal tissue tolerance. This finding highlighted novel treatments where extremely heterogeneous dose delivery in temporal domain can create. There is another way of delivering highly heterogeneous dose distributions with much longer history of practice namely spatially fractionated radiotherapy (SFRT) (Mohiuddin *et al* 1999, Kaiser *et al* 2013, Kanagavelu *et al* 2014). In this case heterogeneous distribution is in spatial domain and it is often reported to successfully treat extremely large tumors difficult to manage with a conventional radiotherapy. The mechanism of SFRT is presumably related to the tumoricidal effect on the untreated region as well as the treated part of tumors, known as the bystander effect (Ngwa *et al* 2018, Barsky *et al* 2019, Bitran 2019, Leary *et al* 2019, Wani *et al* 2019, Yilmaz *et al* 2019, Hatten *et al* 2022). The improved therapeutic gain from such extreme spatio-temporal modulations in dose distribution may need deeper understanding of radiation interactions with cells in physical, chemical and biological levels.

Immune response is believed to play an important role in many aspects of the radiotherapy. Complete tumor regression is often observed with much less dose to kill all cancer cells suggesting radiation may trigger other tumoricidal mechanisms. Slone *et al* 1979 reported that 1.67 time higher dose is required for tumor control of immuno-deficient mice compared to immuno-competent mice. Immune checkpoint inhibitors show outstanding results in a variety of cancers and combinations with radiotherapy are often desired for the synergistic effect for long lasting responses (Vaage 1973, Markovsky *et al* 2019, Davidson *et al* 2022). Thus it is crucial to develop a mechanistic model describing the dynamics of radio-immune response in order to understand the mechanism and efficiently develop clinical studies in this rapidly evolving area (Serre *et al* 2016, Geng *et al* 2017, Asperud 2020, Bekker *et al* 2022).

Radiation not only produces direct cytotoxic effects on the irradiated tumor but also modifies the micro-environment which can influence neighboring cells. Immunogenic cell death by radiation secondarily causes production and release of cytokines and chemokines into the tumor micro-environment followed by the infiltration of dendritic cells to the site of the tumor, activation of dendritic cells and up-regulation of cytotoxic T-lymphocytes. These responses lead to increased levels of anti-tumor immunity and induce damages to the untreated neighboring cells interconnected to the treated tissues (bystander effects). Tissue responses may also relate to other factors in addition to the cytotoxic effects of radiation, however. Tumor heterogeneity in term of cell cycle (Short *et al* 2003), oxygen tension (Kopecka *et al* 2021) and cell-intrinsic genomics (Scott *et al* 2017) are among other important variables. The vascular damage during radiation treatment can play a critical role in determining the level of biological effect. Therefore, some radiosensitizers may improve the bystander effect by blocking cells in radiosensitive phase or regularizing vasculature structures for better oxygenation. Dose fractionation is widely utilized in clinical applications to allow recovery of the normal tissues from radiation damage. The focus of radiobiological study has been shifted from the improvement of therapeutic ratio by changing fractionation schemes towards the characterization of cell cycle kinetics, DNA damages and the mechanism of cell death with the advent of modern molecular biology (Marín *et al* 2015, Scott *et al* 2017, Yilmaz *et al* 2019).

Jeong *et al* (2017) studied a mathematical model to quantitatively understand tumor cellular dynamics during a course of radiotherapy. The model fits outcomes for early stage localized non-small cell lung cancers (NSCLC) assuming a constant local supply of oxygen and glucose, sub-lethal damage, the impact of hypoxia and proliferation. The model however did not include immune response in the cell. Geng *et al* (2017) develop a mathematical model to predict Kaplan–Meier survival curves for chemo-radiotherapy in NSCLC patients. Biological response of tumors to radiotherapy and its immunomodulatory properties were investigated using sets of differential equations by Bekker *et al* (2022) in an attempt to answer questions regarding the timing and dose fractionation for the improved anti-tumor immune responses. Cyclic behavior of tumor cells and lymphocite effect cells was demonstrated using the phase plane analysis in their model.

To address the bystander effect by immunomodulatory responses, Serre *et al* (2016) developed a discrete-time numerical model of radio-immuno-therapy in which radiation is prescribed with inhibitors of the PD1-PDL1 axis and/or CTLA4. This model offered an explanation for the reported biphasic relationship between the size of a tumor and its immunogenicity measured by the bystander effect. It also explained why discontinuing immunotherapy may result in tumor recurrence. In this model, tumor antigens are assumed to be released by tumor cells naturally at a constant rate and at higher rate after radiation exposure. Primary immune response is developed by immune effector cells (tumor infiltrating cytotoxic T lymphocytes (CTLs) or tumor associate macrophages, etc) proportionally generated by the volume of tumor antigens. Immune memory effect learned from the primary immune response was also modelled as a secondary immune response. It was demonstrated that the model can be successfully applied to simulate cancer immunotherapy and the synergy with radiotherapy. The Bystander effects previously reported in the experiment on mice (Vaage 1973) was explained using the model.

Since the immune response is proportional to the irradiated volume in Serre’s model and partial irradiation in SFRT reportedly generates even larger immune response than full irradiation (Mohiuddin *et al* 1999, Kaiser *et al* 2013, Kanagavelu *et al* 2014, Markovsky *et al* 2019, Asperud 2020, Asperud *et al* 2021), Asperud *et al* (Asperud 2020, Asperud *et al* 2021) developed a new numerical model with SFRT in mind. Instead of CTL generation directly from tumor antigens which are not killed by radiation in Serre’s model, Asperud assumed that CTLs need to be activated from a naturally inactive status. The rate of CTL activation was modelled as a function of irradiated tumor volume. The model also included time delay of cell clearance using a ‘doomed cell’ compartment for better agreement with *in vivo*. Damaged cells doomed to die by radiation and immunogenic damage are reportedly not removed from tissues instantly but can take days to be cleared (Lim *et al* 2008, Zhong and Chetty 2014). The model was validated using previously published data on syngeneic xenografts (67NR breast carcinoma and Lewis lung carcinoma). This model, however, did not include the immune suppression effect of tumor cells and the immune boost by immunotherapy agents. In this study, we propose a new model to combine the strengths of two previous models; the effect of tumor volume and immunotherapy inhibitors from Serre’s model and the mechanism of active/inactive CTL and doomed cells in Asperude’s.

The rest of the manuscript is structured as follows. The first section will describe the development of the mathematical tumor model for radio-immune response. In the following sections, analytic solutions of the tumor growth are presented in distinctive boundary conditions distinguished by the bifurcation threshold; immune limited (trapped) versus immune escape. The pattern of tumor growth changes drastically around this bifurcation threshold. Mouse experiment on syngeneic xenografts (67NR breast carcinoma) by Asperud *et al* (2021) and a single case of patient data were used for an application of the developed model.

## 2. Methods

### 2.1. Radio-immune response model

**Figure 1.**
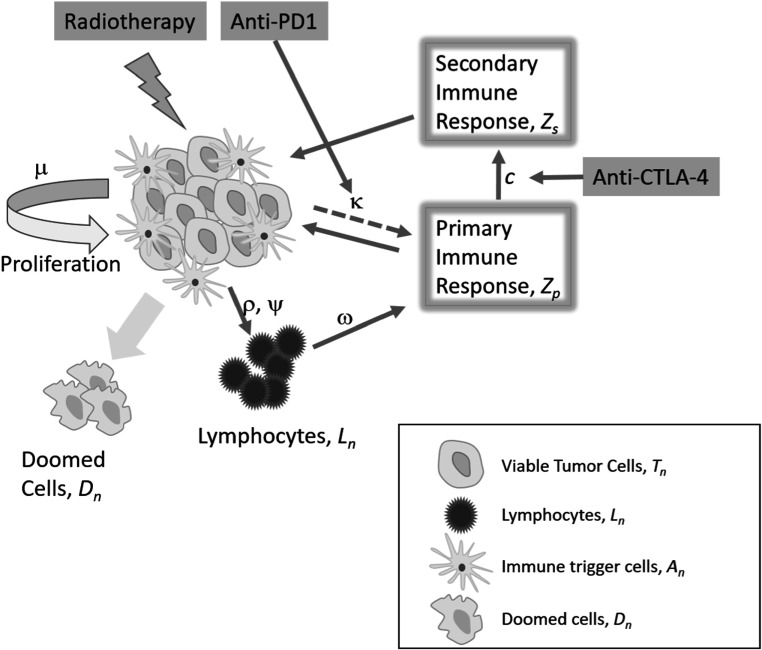
Schematic of the tumor model. Viable tumor cells proliferate exponentially with growth rate of *μ*. Tumor cells lethally damaged by either radiation or immune response turn into doomed cells to be cleared from the body. Lymphocytes are activated by various mechanisms including tumor antigen release in natural and radiation damaged tumor cells. Lymphocytes attack tumor cells by immune response and tumors fight back through an immune suppression mechanism. Immunotherapy can affect the immune responses.

The tumor model in this study consists of four compartments: the volume of viable cancer cells *T*_*n*_, doomed cells *D*_*n*_ which represents dead cells to be removed from the host body with some time delay after lethal damage, active cytotoxic T lymphocytes (CTLs) *L*_*n*_ responsible for the anti-tumor immune response and the density of immune triggering cells *A*_*n*_ converting naive CTLs to be active on attacking tumor cells. The measure of time *n* is expressed as days for all compartments. The schematic is shown in figure 1

\begin{eqnarray*}\begin{array}{rcl}\mathrm{Viable}\ \ \mathrm{tumor}\ \ \mathrm{cells}:\ {T}_{n+1} &amp; = &amp; {T}_{n}\ {e}^{(\mu -{Z}_{n})}\ {S}_{T}\\ \mathrm{Lymphocites}:\ {L}_{n+1} &amp; = &amp; (1-{\lambda }_{L}){S}_{L}\ {L}_{n}\ +\rho \ {T}_{n}+\psi \ \epsilon \ {A}_{n}{T}_{n}\\ \mathrm{Triggering}\ \ \mathrm{cell}\ \ \mathrm{density}:\ {A}_{n+1} &amp; = &amp; (1-\epsilon )\ ({A}_{n}\ {S}_{i}+{\mathrm{\Delta }}{A}_{n})\\ \mathrm{Activation}\ \ \mathrm{function}:\ \epsilon &amp; = &amp; \tanh ((1-{S}_{T}){V}_{c})\\ \mathrm{Primary}\ \ \mathrm{Immune}\ \ \mathrm{response}:\ {Z}_{p,n} &amp; = &amp; \displaystyle \frac{{{wL}}_{n}}{1+\tfrac{\kappa {{T}_{n}}^{2/3}{L}_{n}}{1+{p}_{n}}}\\ \mathrm{Secondary}\ \ \mathrm{Immune}\ \ \mathrm{response}:\ {Z}_{s,n} &amp; = &amp; \displaystyle \sum _{i=0}^{n}\gamma \displaystyle \frac{1+{c}_{i}}{r+{c}_{i}}{Z}_{p,i}\\ \mathrm{Doomed}\ \ \mathrm{cells}:\ {D}_{n+1} &amp; = &amp; (1-{\lambda }_{D}){D}_{n}+(1-{S}_{T})\ {T}_{n}{e}^{\mu }+{S}_{T}\ {T}_{n}\ {e}^{\mu }(1-{e}^{-{Z}_{n}}),\end{array}\end{eqnarray*}

where total immune response is the sum of the primary and secondary immune effect *Z*_*n*_ = *Z*_*p*,*n*_ + *Z*_*s*,*n*_. Parameters and numerical values used in this study are summarized in table 1.

**Table 1.** Summary of model parameters.

	Description	Figure 2	Figure 3	Figure 4	Figure 5	Figure 6
${\left(\alpha ,\beta \right)}_{T}$	Radiation sensitivity of tumor cells, T	NA	^a^	^a^	^a^	^b^
${\left(\alpha ,\beta \right)}_{L}$	Radiation sensitivity of lymphocytes, L	NA	^c^	^c^	^c^	^c^
*μ*	Tumor growth rate	0.217	0.217	0.217	0.217	0.03
*ρ*	Tumor infiltration rate	0.1	0.5	0.5	0.5	0.1
*w*	Rate of cell killing	0.05	0.135	0.135	0.135	0.004
*λ*_*D*_	Decay constant of D	0.039	0.045	0.045	0.045	0.045
*λ*_*L*_	Decay constant of L	0.335	0.045	0.045	0.045	0.056
*λ*_*A*_	Recovery constant of A	0.039	0.045	0.045	0.045	0.045
*ψ*	Radiation induced infiltration	NA	NA	300	300	4.6
		0.000	0.00				
*κ*	Immune suppression effect	0.011	0.50	1.1	1.1	0.5
		0.012	0.51				
		0.013	0.55				

*μ*= 0.217 (T pot = 3.2 d) Asperud *et al* (2021), 0.03 (23 d) fitted to patient data.

Decay (recovery) constant of 0.039 (half life = 17.5 d, Zhong and Chetty (2014)), 0.045 (15 d, Lim *et al* (2008)), 0.056 (12 d, fitted to patient data) and 0.335 (1.7 d, De Boer *et al* (2003)).

^a^ ${\left(\alpha ,\beta \right)}_{T}$ = (0.05, 0.0114) for 67NR breast carcinoma, Asperud *et al* (2021).

^b^ ${\left(\alpha ,\beta \right)}_{T}$ = (0.214, 0.0214) for radioresistant tumor, Gholami *et al* (2016).

^c^ ${\left(\alpha ,\beta \right)}_{L}$ = (0.182, 0.143) Asperud *et al* (2021).

**Viable Tumor Cells**: the growth rate of tumor *μ* is a small positive constant, which is decided by the doubling time, *T*_pot_ following the formula that $\mu ={\mathrm{ln}}(2)/{T}_{{\mathrm{pot}}}$. In the absence of immune response (*Z*_*n*_ = 0), the tumor will grow exponentially. This simplification has been adopted previously for the experimental results on small size tumors (Serre *et al* 2016, Asperud 2020). Although Steel and Lamerton (1966) thoroughly discussed the limitations of exponential growth model in human cancer and other sophisticated tumor dynamics can be considered such as the Gompertz model (Gompertz 1833), such simplification should be adequate for larger tumors since the improvement of the modelling accuracy based on the tumor volume effect such as (vasculature, oxygen, nutrients and metabolism) might be modelled with the immune dynamics (Gerlee 2013). Specific consideration of these dyamics, such as vascular organization could be considered (Scott *et al* 2016), but for this first investigation, we will consider only a homogeneously vascularized tumor.

The immune response *Z*_*n*_ is assumed acting as an anti-tumor growth factor which slows down the tumor growth or even shrinks the tumor volume depending on its intensity. The surviving fraction (*S*) from irradiation is based on the generalized linear-quadratic (LQ) model defined as $S={e}^{-\alpha {d}_{n}-\beta {d}_{n}^{2}}$ where (*α*, *β*) is radiation sensitivity and *d*_*n*_ is radiation dose. The surviving fraction for tumor, CTLs and immune triggering cells are expressed with *S*_*T*_, *S*_*L*_ and *S*_*i*_, respectively.

**Lymphocytes**: there is a growing body of evidence indicating radiation-mediated T-cell priming through the activation of host immunity (Lee *et al* 2009, Victor *et al* 2015, Walle *et al* 2018, Davidson *et al* 2022). Tumor antigens released either from tumors in natural condition or from radiation damaged tumors lead to the priming of tumor antigen specific T-cells. A positive feedback loop is often established by another antigens released from the tumors attacked by the T-cells. Radiation, therefore, may provoke dendrite cell mediated tumor antigen specific T-cell priming, transforming a tumor into an *in situ* vaccine (or create vaccine instead) (Walle *et al* 2018). The finite life span of CTLs is modelled by the decay constant ${\lambda }_{L}=1-{e}^{-{ln}2/{T}_{L}}$, where *T*_*L*_ is the half life of CTLs and by radiation surviving fraction *S*_*L*_. The naive CTLs are activated in natural conditions proportional to the tumor volume. This is modelled by tumor infiltration rate, *ρ*. The CTL activation can be further amplified proportionally (at the rate of *ψ*) to the radiation-damaged tumor cells, *ϵ* *T*_*n*_ and the amount of immune triggering cells (such as dendrite cells attracted by ATP released from tumors), *A*_*n*_. The immune triggering cells is modelled to be 1.0 in fully saturated conditions before radiation and can be reduced by radiation damage determined by surviving fraction *S*_*i*_. The rate of CTL activation *ϵ* depends on the relative volume of radiation-damaged cancer cells, *V*_*c*_. We use the hyperbolic tangent to limit the range between 0 and 1. The CTL activation from radiation-damaged tumors is thus completed by *ψ* *ϵ* *A*_*n*_ *T*_*n*_.

**Triggering cell density**: immune triggering cell density *A*_*n*_ is reduced at the rate of *ϵ* after the activation of CTLs and can be damaged by radiation. Surviving fraction of immune triggering cells is denoted by *S*_*i*_. For long-term analysis such as re-treatment cases taking months or years, *A*_*n*_ can be further assumed to be slowly saturated back after the radiation damage Δ*A*_*n*_ = (1 − *A*_*n*_)*λ*_*A*_, where *λ*_*A*_ is the recovery constant similarly defined as *λ*_*L*_.

**Immune response**: the primary anti-tumor immune response is assumed proportional to the amount of CTLs with the rate of cell killing, *w* when there is no immune suppression effect, *κ* = 0. It is known that the immune response is tempered by tumor cells proportional to the surface area of tumor volume and the amount of CTLs, *L*_*n*_. Thus immune suppression effect is modelled by $\kappa {T}_{n}^{2/3}{L}_{n}$ (Serre *et al* 2016). Immunotherapy can modify the immune suppressive capability of cancer cells. In our primary immune response equation, *p*_*n*_ is the effect of PD1-PDL1 immunotherapy to control the tumor’s immune suppression. *Z*_*s*_ is the secondary immune response or memory effect learnt from primary immune reaction, *γ* is the sensitivity factor for *Z*_*s*_, *r* is the normalization factor and *c*_*n*_ is the effect of CTLA4 immunotherapy. A detailed explanation on immunotherapy effect can be found in the literature (Serre *et al* 2016).

**Doomed cells**: after lethal damage, it can take up to 1–2 weeks to clear these cells from the body. Therefore *in vivo* tumor volume measurement may not correlate well with viable tumor cells. Sum of viable tumor cells and doomed cells may be related better with the measurement (Asperud *et al* 2021). The volume of damaged CTLs and immune triggering cells are not included in the doomed cell formulation in this analytic model for simplicity. Decay constant of doomed cell *λ*_*D*_ is expressed in a similar way as *λ*_*L*_.

The amount of CTLs in the formulation can be reduced asymptotically when *ϵ* is small.

\begin{eqnarray*}\begin{array}{rcl}{L}_{n+1} &amp; = &amp; (1-{\lambda }_{L})\ {S}_{n}\ {L}_{n}+\rho \ {T}_{n}+\psi \ \epsilon \ {A}_{n}{T}_{n}\\ &amp; \simeq &amp; (1-{\lambda }_{L})\ {S}_{L}\ {L}_{n}+\rho {T}_{n}+\psi (1-{S}_{T}){V}_{c}\ {A}_{n}{T}_{n}.\end{array}\end{eqnarray*}

The delay between radiation damage and the death of tumor cells is modelled using an aggregated survival probability model (Serre *et al* 2016). In this study, the same mean of 3 d and the standard deviation of 1.5 d were used in a log-normal density probability function which translates 95% of lethally damaged cells died in 6 d after radiation.

**Treatment planning**: immune response of tumor cells depends on the amount of radiation dose. In order to consider the effect of heterogeneous dose distribution, the mathematical model should be applied to each subsection of tumor volume which has the same dose level. Total tumor volume can be measured as the sum of each subsection of tumor volume. Dose distribution can range from the simple case of two-level (from open field versus blocked field) to the multi-level dose spectrum often found in clinical cases for cancer treatment. To correctly account for such heterogeneous dose distribution, dose volume histogram was extracted from the treatment planning system. Mathematical model was applied to each voxel.

### 2.2. Boundary behavior

There exist distinct phases dictated by boundary conditions in the proposed numerical model. Some of the fundamental phases are reviewed in this section in order to understand the mechanism of radio-immune dynamics. Although the model is capable of considering immunotherapy drugs, for simplicity, we do not consider these in the boundary behavior analysis. The secondary immune response is also neglected in the analysis and simulations.

#### 2.2.1. Case 1: immune limited

One of the simple boundary conditions would be tumors with the lack of secondary immune capability (*γ* = 0), inability of immune suppression effect (*κ* = 0), no use of immunotherapy drugs (*p* = 0) and no radiation (all *S* = 1). In this case, the tumor will start to grow exponentially at the initial phase and the growth rate slows until it stops growing completely at the equilibrium condition (*Z*_*n*_ = *μ*). The anti-tumor immune response, *Z*_*n*_ is linearly increasing with the tumor volume in this condition. The terminal viable tumor volume, *T*_∞_ in the equilibrium condition can be found with *L*_*n*+1_ ≃ *L*_*n*_ and *S*_*L*_ = *S*_*T*_ = 1 in equation (2)

\begin{eqnarray*}\begin{array}{l}\,{Z}_{n}=w\ {L}_{n}=w\displaystyle \frac{\rho }{{\lambda }_{L}}{T}_{n}=\mu \\ \quad \therefore \quad {T}_{\infty }=\mathop{\mathrm{lim}}\limits_{n\,\to \infty }{T}_{n}=\displaystyle \frac{{\lambda }_{L}}{w\rho }\ \mu .\end{array}\end{eqnarray*}

The amount of doomed cells in the equilibrium condition can be found similarly as shown below

\begin{eqnarray*}\begin{array}{l}\,{D}_{n}=\displaystyle \frac{{T}_{n}}{{\lambda }_{D}}{e}^{\mu }(1-{e}^{-{Z}_{n}})\\ \quad \therefore \quad {D}_{\infty }=\mathop{\mathrm{lim}}\limits_{n\,\to \infty \,}{D}_{n}=\displaystyle \frac{{T}_{\infty }}{{\lambda }_{D}}({e}^{\mu }-1).\end{array}\end{eqnarray*}

This equilibrium condition is an example of an immune limited, likely pre-malignant tumor that requires immune escape to grow further (one of the hallmarks of cancer, Weinberg and Hanahan (2000), Hanahan and Weinberg (2011)).

#### 2.2.2. Case 2: immune escape and bifurcation condition

When immune suppression becomes larger than a certain threshold ($\kappa > \hat{{\varepsilon }_{k}}$) with all other conditions the same as Case 1, tumors can break the equilibrium and keep growing (immune escape). In this condition, the volume of active CTLs also keep growing with the tumor volume. The bifurcation threshold to break the equilibrium, $\hat{{\varepsilon }_{k}}$ can be found as shown below

\begin{eqnarray*}\begin{array}{c}\,{Z}_{n}\,=\,\displaystyle \frac{{wL}_{n}}{1+\kappa {T}_{n}^{2/3}{L}_{n}}\,< \mu \\ \kappa \,> \,\displaystyle \frac{w}{\mu {T}_{n}^{2/3}}-\displaystyle \frac{1}{{L}_{n}{T}_{n}^{2/3}}={\varepsilon }_{k}\\ \,\therefore \,\kappa \,> \,\hat{{\varepsilon }_{k}}=max({\varepsilon }_{k}).\end{array}\end{eqnarray*}

The maximum *ε*_*k*_ is found at ${T}_{n}=\tfrac{5\ \mu {\lambda }_{L}}{2(1-\mu )\ w\rho }$ or ${T}_{n}=\tfrac{2.5}{1-\mu }{T}_{\infty }$ from the solution of $\tfrac{\partial }{\partial n}{\varepsilon }_{k}=0$ with the approximation of equation (2) such that ${L}_{n}=\tfrac{\rho }{{\lambda }_{L}}{T}_{n}-\tfrac{{\mathrm{\Delta }}{L}_{n}}{{\lambda }_{L}}\simeq \tfrac{\rho (1-\mu )}{{\lambda }_{L}}{T}_{n}$ for Δ*L*_*n*_ ≃ *ρ*Δ*T*_*n*_ = *ρ* *μ* *T*_*n*_ in early phase of growth where tumor growth dominates the immune reaction and breaks equilibrium condition. The bifurcation threshold therefore can be found as

\begin{eqnarray*}\hat{{\epsilon }_{k}}\simeq \alpha \displaystyle \frac{w}{\mu {T}_{\infty }^{2/3}},\end{eqnarray*}

where $\alpha ={\left(\tfrac{1-\mu }{2.5}\right)}^{2/3}\left(\tfrac{1.5+\mu }{2.5}\right)$. As will be discussed later, equation (6) is an approximated solution for the bifurcation threshold, it agrees well for stably transient cases but tends to over-estimate for unstable transient cases where both immune response and immune suppression are very strong. In other words, tumors with highly unstable transient dynamics may escape the equilibrium condition earlier than the approximated solution of the bifurcation threshold in equation (6).

#### 2.2.3. Case 3: generalized expression

Immune effect *Z*_*n*_ can be rewritten by substituting *κ* with $\hat{{\varepsilon }_{k}}(1+\delta )$, where *δ* ≥ −1. With this representation, Case 1 and 2 can be considered as a special case of the general representation with *δ* = −1 for Case 1 and *δ* = 0 for Case 2. Tumors can be in one of the conditions below

\begin{eqnarray*}\begin{array}{l}{Z}_{n}=\displaystyle \frac{{{wL}}_{n}}{1+\hat{{\varepsilon }_{k}}(1+\delta ){T}_{n}^{2/3}{L}_{n}}=\mu \ (\mathrm{Equilibrium})\\ \,< \,\mu \ (\mathrm{Imune}\ \ \mathrm{escape})\\ \,> \,\mu \ (\mathrm{Immune}\ \ \mathrm{limited}).\end{array}\end{eqnarray*}

The equation above can be represented by substituting *κ* with $\hat{{\varepsilon }_{k}}(1+\delta )$ using equation (6). Tumors at the condition of immune limited is written below

\begin{eqnarray*}{{wL}}_{n}\left(1-\alpha (1+\delta ){\left(\displaystyle \frac{{T}_{n}}{{T}_{\infty }}\right)}^{2/3}\right)\ > \mu .\end{eqnarray*}

The left side of equation (7) can be considered as a general form of the immune effect, *Z*_*n*_:

\begin{eqnarray*}{Z}_{n}={{wL}}_{n}\left(1-\alpha (1+\delta ){\left(\displaystyle \frac{{T}_{n}}{{T}_{\infty }}\right)}^{2/3}\right).\end{eqnarray*}

As previously described, equation (8) is reduced to Case 1 and 2 with *δ* = −1 and *δ* = 0, respectively. For tumor with immune escape capability, therapeutic intervention is needed to control the tumor volume. Radiotherapy can kill tumor cells not only by radiation damage but also by boosting CTL generation through the process expressed by the term of *ψ* *ϵ* *A*_*n*_ *T*_*n*_ in equation (2). To keep the tumor volume under control, it is necessary to have the parenthesis in equation (8) a positive value. The condition is translated that the radiation treatment should start early enough before the tumor volume reaches the critical tumor volume shown below

\begin{eqnarray*}{T}_{n}< {\left(\displaystyle \frac{1}{\alpha (1+\delta )}\right)}^{3/2}{T}_{\infty }.\end{eqnarray*}

The generalized terminal viable tumor volume, *T*_∞,*δ*_ can be found from the solution of *Z*_*n*_ = *μ* in equation (8) with Taylor expansion of *T*_*n*_/*T*_∞_

\begin{eqnarray*}{T}_{\infty ,\delta }\simeq \displaystyle \frac{9-22\alpha (1+\delta )}{9-25\alpha (1+\delta )}{T}_{\infty }.\end{eqnarray*}

The terminal viable tumor volume in Case 1, *T*_∞_ is a special case with *δ* = −1.

## 3. Results

**Figure 2.**
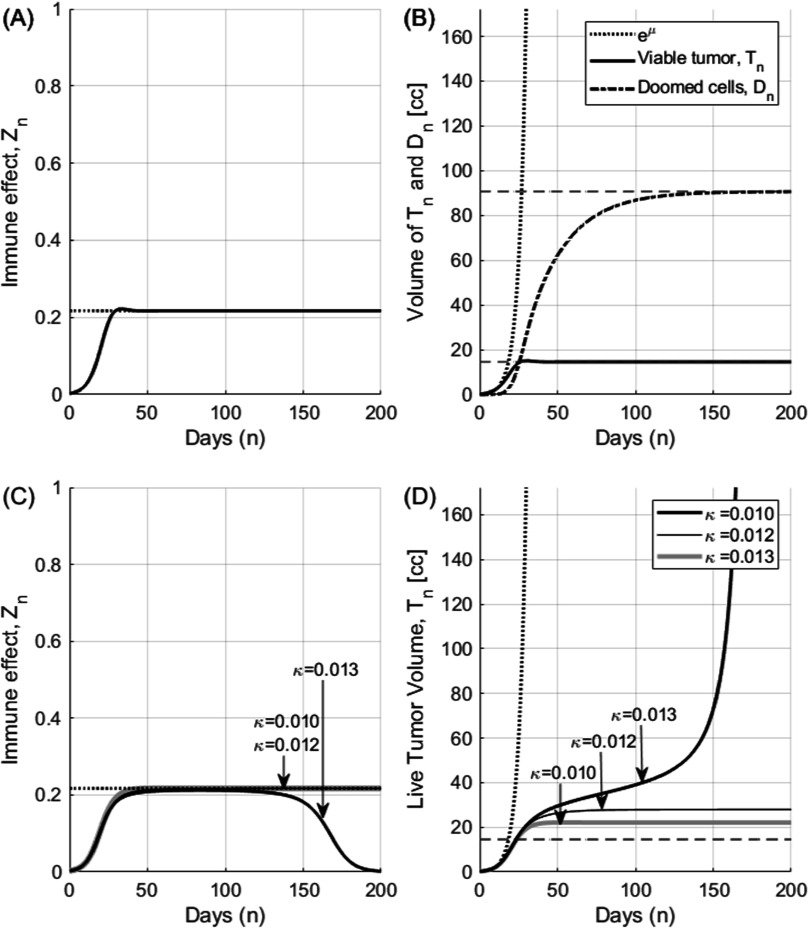
Dynamics of tumor immune effect, terminal viable tumor volume (Case 1) and the bifurcation of tumor growth (Case 2). It is assumed that *μ* = 0.217 (*T*_pot_ = 3.2 d), *ρ* = 0.1, *w* = 0.05, *λ*_*D*_ = 0.039 (half life of 17.5 d) and *λ*_*L*_ = 0.335 (half life of 1.7 d). Detailed model parameters can be found in table 1. (A) With no immune suppression (*κ* = 0), immune effect *Z*_*n*_ reaches the equilibrium status equal to the tumor growth rate, *μ* depicted with a dashed horizontal line. (B) Viable tumor cells *T* and doomed cells *D* converge to the estimated terminal volume of 14.5 c.c. and 90.7 c.c. as shown in horizontal dashed lines calculated with equation (3) and (4). Exponential tumor growth is shown in the dotted line as a reference in case of no immune effect or complete immune suppressed subjects. (C) Immune effect, *Z*_*n*_ starts suffering from immune suppression (*κ* > 0.0). It is hindered completely when *κ* is larger than the bifurcation threshold ($\hat{{\epsilon }_{k}}=0.0123$ in this example). (D) The terminal viable tumor volume, *T*_∞,*δ*_ is increasing with *κ* as shown in equation (10). The horizontal broken line shows the terminal viable tumor volume at *κ* = 0 in Case 1 (*T*_∞_) for comparison. Tumor can grow out of the equilibrium condition and enters into the immune escape mode when *κ* is larger than the bifurcation threshold.

We analyzed our novel model of radio-immuno response and found two distinctive boundary conditions as described in the previous sections. In figures 2(A) and (B) we show the terminal tumor volume with potential doubling time of 3.2 d (67NR mice data, Markovsky *et al* (2019)) for an illustration of the Case 1 (*κ* = 0.0). The primary immune effect and the decay constant of CTL are modeled with *ρ* = 0.1, *w* = 0.05, *λ*_*D*_ = 0.039 (half life of 17.5 d) and *λ*_*L*_ = 0.335 (half life of 1.7 d), respectively (Asperud 2020, Asperud *et al* 2021). Full model parameters can be found in table 1. The immune effect *Z*_*n*_ is increasing with tumor volume and reaches the equilibrium status equal to the tumor growth rate, *μ* (figure 2(A)). The tumor volume *T*_*n*_ and the doomed cell volume *D*_*n*_ converge well to the estimated terminal volume of 14.5 c.c. and 90.7 c.c., respectively as shown in horizontal dash lines in figure 2(B) calculated with equations (3) and (4). The dynamics of the immune effect, *Z*_*n*_ looks like that of the viable tumor volume but with some time lag about the half life of lymphocyte, *λ*_*L*_. An exponential tumor growth is shown in the dotted line as a reference when no primary immune reaction exists in the cells or immune suppressed subjects are considered. Tumors with immune suppression capability (*κ* > 0) can grow larger than those without the capability as shown in figures 2(C) and (D). As immune suppression is larger, immune effect suffers and is eventually suppressed below the tumor growth rate, *μ*. This leads to catastrophic tumor growth, called bifurcation condition as described in equation (6). The terminal tumor volume at the equilibrium, *T*_∞,*δ*_ increases with immune suppression effect, *κ* (or equivalently *δ*) as expected in equation (10)and as shown in figure 2(D). The maximum viable tumor volume in the immune limited mode is 27.9 c.c. well within the bifurcation limit of 30.9 c.c. calculated from equation (9).

**Figure 3.**
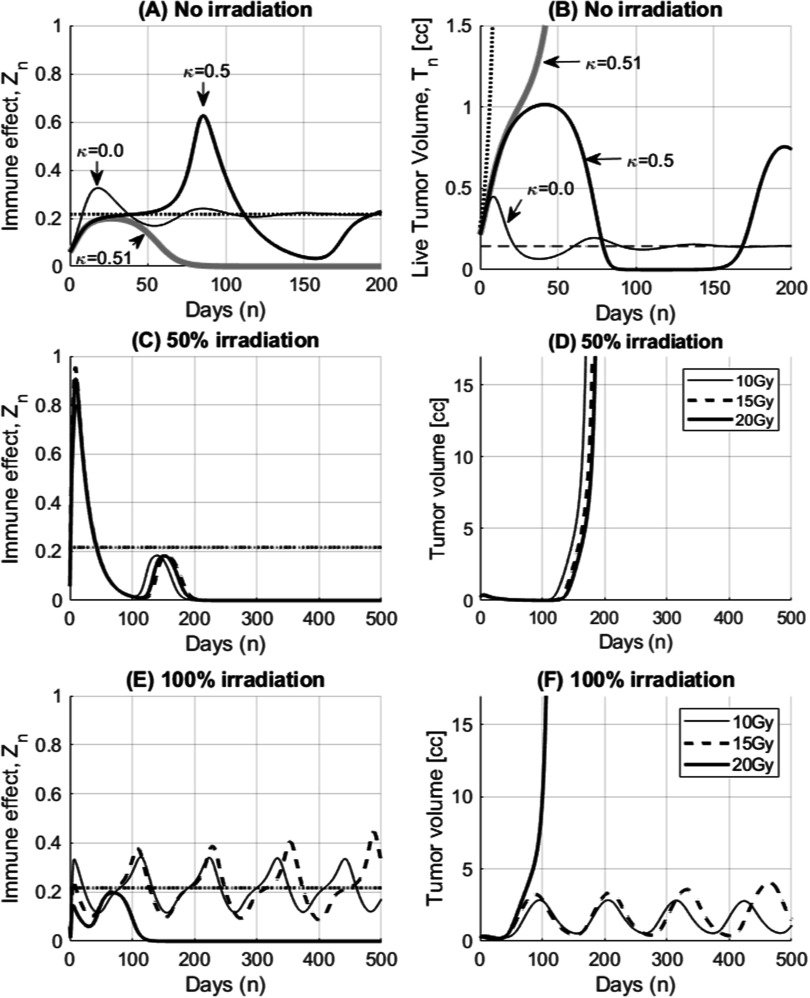
Dynamics of tumor immune effect and the bifurcation of tumor growth. In this example, the same tumor doubling time but much stronger immune reaction (larger *μ*, *w* and smaller *λ*_*L*_, *λ*_*D*_) is assumed. (A) Immune effect shows unstable dynamics over time. The magnitude of instability increases with the immune suppression effect, *κ*. When *κ* becomes larger than certain level (0.51 in this case) however, the immune effect is suppressed most of the time and the tumor can change its mode to the immune escape (continuous tumor growth). (B) The terminal tumor volume *T*_∞_ is 0.15 c.c. shown in the horizontal dash line which is only about 10% of Case 1 (figure 2) due to the stronger immune reaction. The bifurcation occurs at *κ* = 0.51 (early immune escape), smaller than the solution of 0.72 from equation (6). In (C)–(F) *κ* is set to 0.55 which is an early immune escape condition with some margin but still less than the bifurcation threshold of 0.72. Partial irradiation with 20 Gy triggers larger immune reaction (C) compared to full irradiation (E) with 20 Gy. When adequate amount of dose (1 Gy ≤ dose ≤ 10 Gy in single fraction) is given to the whole tumor volume in (E) and (F), the immune effect becomes similar to the level of tumor growth factor (arrested). Partial volume irradiation does not show such arrest event. Tumor volume in vertical axis represents *T*_*n*_ + *D*_*n*_.

Tumors with stronger immune reactions can result in a smaller terminal tumor volume but the change in tumor volume becomes more dynamic. As an example, tumors with the same doubling time of 3.2 d but with much stronger immune response of *ρ* = 0.5, *w* = 0.05, *λ*_*D*_ = 0.045 (half life of 15 d) and *λ*_*L*_ = 0.045 (half life of 15 d) are considered in figure 3. Larger infiltration rate *ρ* and longer half life of CTLs *λ*_*L*_ represent a stronger immune reaction. The terminal viable tumor volume *T*_∞_ is 0.15 c.c. much smaller than 14.5 c.c. in figure 2. The immune effect *Z* reaches the equilibrium status with dynamics akin to an attenuated oscillation due to the competing actions of tumor growth (*μ*) and immune reaction (*ρ* and *w*) for tumor without immune suppression capability, *κ* = 0. The shape of the immune effect, *Z* looks like the shape of the viable tumor volume again with some time lag about the half life of lymphocyte, *λ*_*L*_. This oscillating behavior gets amplified with increasing immune suppression effect, *κ* and the tumor growth finally breaks out of the equilibrium. As shown in figure 3(B), immune escape occurs at *κ* = 0.51 which is smaller than the bifurcation threshold, $\hat{{\epsilon }_{k}}$ of 0.72 calculated from equation (6). The maximum viable tumor volume before bifurcation is 1.02 c.c. well within the limit of 1.39 c.c. from equation (9). The early escape is due to the dynamic characteristics of tumor volume overshoot in this condition. Assumptions used in the derivation of equation (6) such as steady change of status are responsible to this discrepancy. Such complex tumor dynamics when host body’s strong immune system vigorously fights against tumor’s immune suppression becomes more complex when radiation is involved.

The effect of immune response in partial or full irradiation is simulated in (C)–(F). Radiosensitivity (*α*, *β*) of tumor and CTLs is assumed to be (0.05, 0.0114) and (0.182, 0.143), respectively in the unit of [Gy^−1^, Gy^−2^] with *ψ* = 300 and *κ* = 0.55. Note that *κ* is well within early immune escape condition with some margin but still less than the bifurcation threshold of 0.72. Partial irradiation (50% tumor volume) with 20 Gy triggers larger immune reaction (C) at early phase compared to full irradiation (E) with 20 Gy. As a result, the time to tumor re-growth is also larger for partial irradiation in (D) and (F). When adequate amount of dose (1 Gy ≤ dose ≤ 10 Gy in single fraction) is given to the whole tumor volume (full irradiation) in (E) and (F), the immune effect becomes similar to the level of tumor growth factor. As a result tumor volume can stay at the equilibrium condition long period time with some visible oscillation. Since tumor cannot make early immune escape, it can be considered as arrested in the immune limited condition. Partial volume irradiation does not show such arrest event.

**Figure 4.**
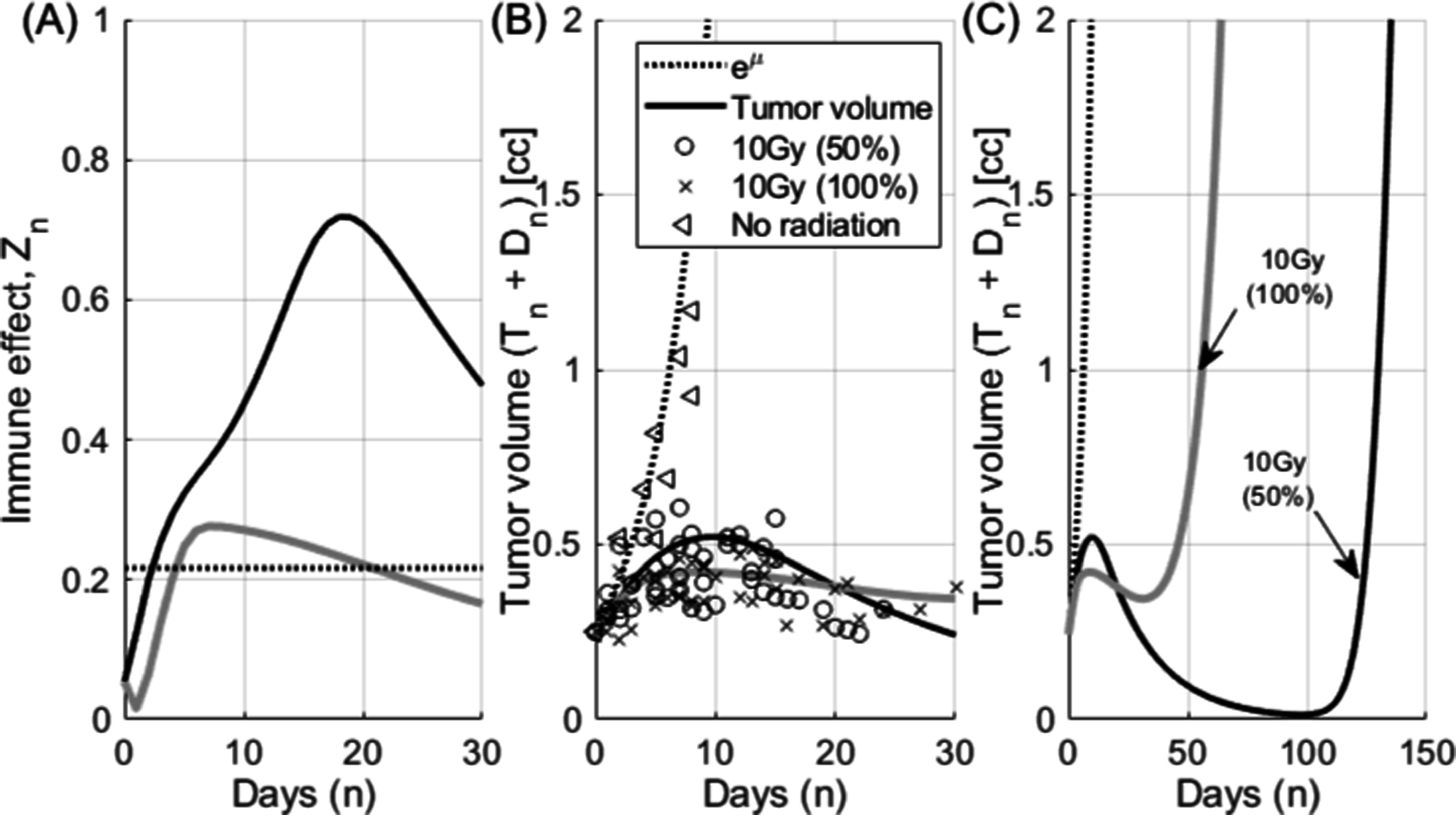
Radio-immune response of 67NR tumors (Adapted from Markovsky *et al* 2019, Copyright (2019), with permission from Elsevier). Tumors (mean volume of 0.25 c.c.) were irradiated with 10 Gy either at 50% (in gray) or 100% coverage (in black) in the experiments. Model parameters including radiation sensitivity are summarized in table 1. The *S*_*i*_ is assumed to be the same as *S*_*L*_. (A) Immune effect is larger for the partial irradiation due to the less radiation damaged immune triggering cells and CTLs. (B) Total tumor volume (*T*_*n*_ + *D*_*n*_) in solid black line is compared with the measurements. The measurement points were lifted digitally (Adapted from Asperud *et al* 2021. © 2021 Institute of Physics and Engineering in Medicine. All rights reserved.). Although cell death by radiation is less for the partial irradiation of 50%, the cell death by immune reaction is larger and longer than full irradiation. Measurement was available up to 30 d in the original study. (C) The growth of tumor volumes are extrapolated up to the range of 150 d.

The effect of immune response in partial or full irradiation on 67NR tumors is simulated in figure 4. All other parameters are the same as in the previous simulation figure 3 except *κ* = 1.1. Total tumor volume (sum of the viable tumor volume, *T*_*n*_ and the doomed cell volume, *D*_*n*_) is compared with the measurement data originally published by Markovsky *et al* and digitally lifted later by Asperud *et al* 10 Gy of radiation is delivered in a single fraction on either 50% (partial) or 100% (full volume) of the tumor volume. Due to the larger radiation damages, the tumor volume is reduced faster in the full irradiation case especially in early phase (<20 d) as shown in figure 4(B). However the less damaged CTLs from the partial irradiation trigger immune reaction in a higher level for longer period (figure 4(A)). As a result, the tumor volume is controlled better at later phase (>20 d) with the partial irradiation as shown in figure 4(B). Simulation result is extrapolated to the range of 150 d in figure 4(C). Although the extrapolated result cannot be validated since the measurement is available only up to 30 d in the original study, the hypothetical behavior of the tumor growth under partial and full irradiation can be reviewed with the model. With much more activated CTLs, the time delay of tumor re-growth is expected greater for the partial volume irradiation.

**Figure 5.**
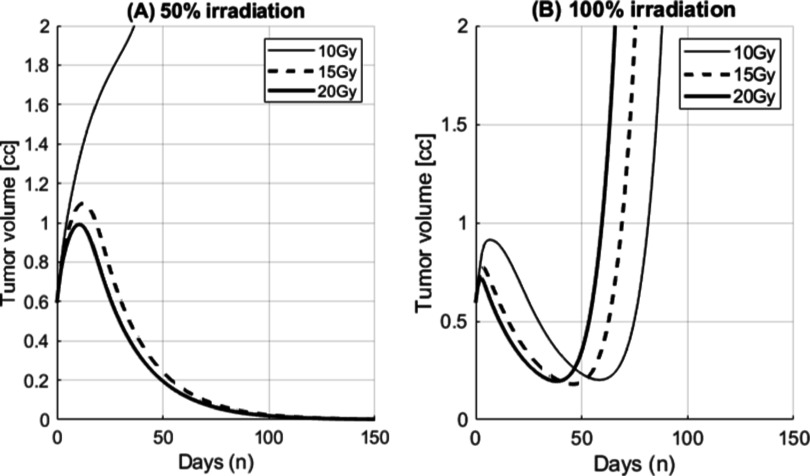
Effect of the tumor volume at treatment (critical tumor volume). In this simulation, tumor was left to grow for 5 extra days before radiation compared to the previous experiment in figure 4. Tumor volume at the time of treatment becomes 0.587 c.c. compared to 0.25 c.c. (A) For the partial irradiation, dose prescription is critical for the success of treatment. When dose is not sufficient (10 Gy), the tumor volume may exceed the critical tumor volume of 1.4 c.c. as described in equation (9) before immune reaction is fully developed. As a result, tumors can keep growing. (B) On the other hand, conventional full irradiation can control the tumor volume with the same dose of 10 Gy. Relatively rapid volume reduction can hold the tumor volume below the critical tumor volume. As a result, conventional full irradiation appears less sensitive to the dose prescription parameters.

The tumor volume at the time of treatment can be critical considering the tumor control condition described in equation (9) and as shown in figure 5. As tumor grows close to its critical volume, there is relatively small time window for successful treatment intervention. Delay in the treatment or not enough dose before the tumor outgrows the critical volume described in equation (9) may lead failure in tumor control. For this simulation, tumor is assumed left to grow for 5 extra days from the previous experiment in figure 4. Tumor volume at the time of treatment is now 0.587 c.c. instead of 0.250 c.c. in figure 4. When dose is not sufficient (10 Gy) in partial irradiation, the tumor can grow over the critical tumor volume of 1.4 c.c. calculated from equation (9) before immune reaction is fully developed (figure 5(A)). On the other hand, full irradiation can control the tumor volume within the critical tumor volume with the same dose of 10 Gy, because relatively rapid volume reduction can occur in early phase. As a result, conventional full irradiation appears less sensitive to the dose prescription in this case. Therefore, special care should be taken if partial irradiation is considered when the tumor volume is approaching the critical volume. Any delay of the treatment could be detrimental. Time delay of tumor re-growth appears still larger with partial irradiation.

**Figure 6.**
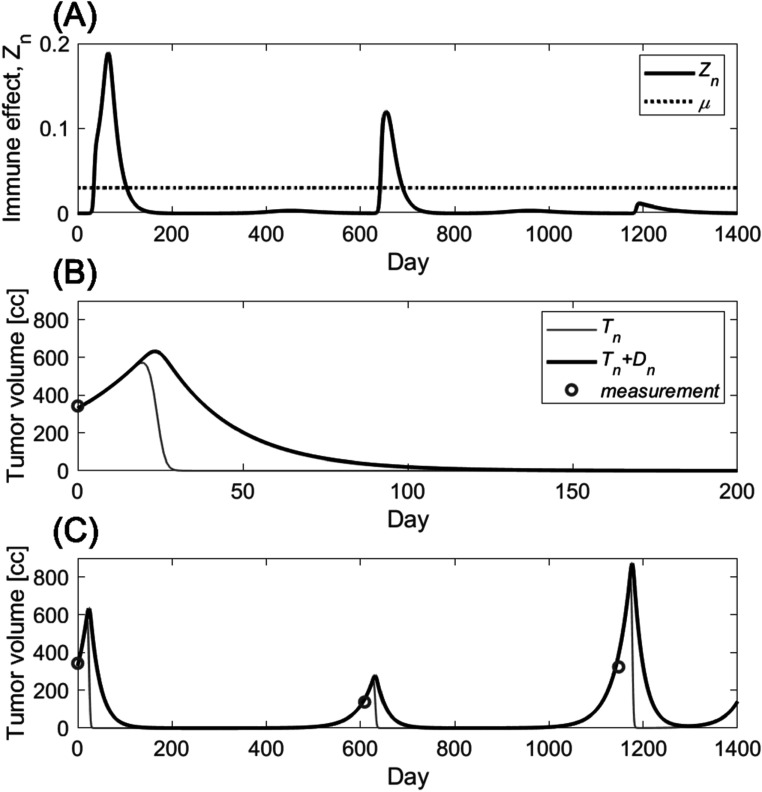
Tumor volume change in a sarcoma patient who received retreatment of radiotherapy. The patient was treated three times over 1200 d with the prescriptions of 5 daily fractions of 6 Gy (The treatment started on day 12), another 5 fractions of 6 Gy (day 609) and 5 fractions of 4 Gy (day 1148). The first fraction of the last treatment session was simultaneous integrated boost of SFRT (15 Gy at max). (A) shows the immune effect triggered by radiation in a solid line and growth rate *μ* in a dotted horizontal line. (B) shows the tumor volume change during the first session of the treatment. The tumor volume (CTV) measured from CT simulation is shown in a circled marker. (C) shows the tumor volume from the model (lines) and the tumor volume measurement from each CT scan (circles).

The model is applied to the tumor volume measurement of a sarcoma patient who received radiotherapy three times over 1200 d; 5 daily fractions of 6 Gy starting on day 12 after the first simulation, another 5 fractions of 6 Gy on day 609 and 5 fractions of 4 Gy on day 1148. The first fraction of the last treatment session was simultaneous integrated boost of SFRT (15 Gy at max). Treatment planning system calculated dose distribution in the patient with the voxel size of 0.4 cm × 0.4 cm × 0.4 cm in this example. The mathematical model is applied to each individual voxel in the tumor volume in order to consider spatially heterogeneous dose distribution. Tumor volume is assumed immobile and immune effect, *Z* is assumed effectively diffused (average immune response was applied to each tumor cells). The immune effect triggered by radiation is shown in figure 6(A) and the tumor volume over the course of treatments are shown in lines with the volume measurement from each CT simulation in circled marks in figures 6(B) and (C).

## 4. Discussion

Understanding the role and response of the immune system during and after radiotherapy is of critical importance as we move further into the era of immunotherapy. It is therefore crucial to develop mechanistic mathematical models of these phenomena to allow us to develop quantitative hypotheses. Deeper understanding of the mechanism may allow to efficiently design clinical studies aiming to amplify the synergistic effect of the radiation and the immunotherapies for cancers. In this study we extend and synthesize two previous models (Serre *et al* and Asperud *et al*). By reformulating the original equations demonstrated that two models are closely related and the effect of partial volume irradiation is explained well by the introduction of CTLs activation.

SFRT is a special radiotherapy technique with long history used before the introduction of conformal planning. It is known to be useful for large tumors difficult to manage with conventional homogeneous treatment planning. Although forgotten for few decades with the introduction of high precision radiotherapy such as IMRT/VMAT, the resurrection of this old technique is recently empowered by IMRT/VMAT (Amendola *et al* 2020, Wu *et al* 2020). Contrary to the collimator-based SFRT where the maximum heterogeneity is at few centimeters depth in tissue, IMRT/VMAT based SFRT can deposit the most heterogeneous dose at deeper part of the body where tumor is located. Despite the increasing interest, therapeutic gain of SFRT enabled by so called *bystander effect* is not yet fully understood. Serre *et al* developed a model to explain biphasic relationship between the size of a tumor and its immunogenicity measured by the *bystander effect*. Markovsky *et al* reported the delay of tumor growth in two groups of mice with 67NR tumor when treated with radiation; one with partial volume and the other with full volume irradiation. Radiation cell killing alone couldn’t explain the measurement data where half volume irradiation shows the same tumor control as full volume irradiation. Asperud *et al* explained that the activation of CTLs is responsible for the development of immune response and full volume irradiation damages not only tumors but also the activated CTLs. As a result radiation cell killing of full volume irradiation may compensate immune response cell killing.

A new mathematical model was proposed in this study combining the strengths of two models. As shown in equation (2) in our mathematical representation, Serre’s model can be considered as a special case of Asperud’s model (either no dose or full dose). Starting from one of the simplest conditions (for cancers without immune suppression capability under no radiation) to the most complex condition (for cancers with fully capable immune suppression effect under radiation), the analysis of our model revealed that there are two distinct modes of tumor response: immune limited versus immune escape and its bifurcation condition. The model is capable to explain the development of immune response and the critical condition that immune response is becoming ineffective. Understanding of complex immune response and those important conditions is important in the successful design of SFRT study. With the CTLs activation at an un-irradiated region, partial irradiation seems to have longer term tumor control at the expense of slower reduction of tumor volume in early phase of treatment as shown in figure 4. Since tumor cell killing by immune response is not effective anymore once the tumor grows beyond the critical volume (equation (9)), SFRT with not enough dose may lose the time window for tumor control as shown in figure 5. In this case, tumor growth reaches the critical volume before immune reaction is fully developed. On the contrary, full irradiation shows more consistent results over large range of dose prescription. In other words, conventional treatment with homogeneous dose distributions seems to rely less on immune reaction. Therefore, it is crucial that deeper understanding of radio immune response is required for the successful applications of SFRT and special care should be taken when partial irradiation is applied if tumor volume is doubted to be close to the critical condition.

The model shows that cancers without immune suppression capability grow exponentially at first and slowly converge to the equilibrium, named as the terminal tumor volume. The tumor growth curves initially exponential and saturating to the equilibrium looks similar to other constraint growth models such as Gompertz model (Gompertz 1833). This represents the generality of the proposed model. The condition of equilibrium, however, does not mean the tumor is not life threatening.

The terminal tumor volume increases with immune suppression capability, *κ*. When *κ* is greater than the certain level called bifurcation threshold, the host body’s immune effect is suppressed below the tumor growth factor, *μ* and the tumors can grow indefinitely out of the terminal tumor volume. If the host body’s immune response is stronger, tumor growth can be limited and the terminal tumor volume is significantly smaller than those with weak immune response. In such case, the body may be able to tolerate the terminal tumor volume with finite size. When both immune response and immune suppression are strong, dynamics of tumor growth becomes unstable and tumor volume swings around the terminal tumor volume as a consequence of the competing immune dynamics.

Due to the dynamic behavior where tumor volume is overshooting, tumors may be able to escape the immune limited condition earlier than the solution in equation (6) and figure 3. As shown in figure 3, tumors capable of early escape can be re-arrested to the immune limited condition when adequate amount of radiation dose is given as low as 1 Gy up to 10 Gy. Dose greater than 10 Gy was not successful because it eradicates not only tumor cells but also depletes CTLs responsible to the development of immune response. As shown in figure 3(E), immune response is depleted below, *μ* all the time. When the dose is too small (less than 1 Gy), it does not attenuate the vigorous dynamic behavior of tumor growth and allows early immune escape. Note that adequate amount of dose makes immune response is hanging around the tumor growth factor. The concept of ‘more dose the better response’ may not be applicable in this case. On the contrary, SFRT in this case did not arrest the tumor to the immune limited mode. Instead boosted immune response at the time of radiation reduced tumor volume effectively but the immune response is forgotten afterward with tumor volume reduction. If not completely eradicated, tumor may grow back.

There are many limitations in this study. The secondary immune response, long term memory effect of immune response was not considered in the simulations for the sake of simplicity and lack of long term data for the validation of this parameter. With the secondary immune effect included, the oscillating behavior of tumor volume in figure 3 may somewhat attenuate and the range of dose for re-arrest in figure 3 could be extended. Model parameters used in this study are mostly based on the mouse study reported in the literature, and, while useful, should be considered only as a starting point, as mouse and human geometries are very different, which would affect percentages of tumor infiltration versus circulating CTLs and therefore the irradiated fraction thereof. A more complete treatment of this could consider formal modeling of the tumor versus circulating fraction, as in Hammi *et al* (2020). Further, for parsimony, a number of important complexities effecting tumor radiosensitivity have not yet been considered. Specifically, it is well known that heterogeneities in oxygen tension can affect both radiation (Scott *et al* 2016) and immunotherapy (Graham and Unger 2018), as well as intratumor heterogeneity in cell type (e.g. stem versus non-stem (Arnold *et al* 2020), and genomics (Scott *et al* 2017)).

Subsequent application of the model to human patients should be considered carefully after thorough mathematical/computational study on the model parameters based on sophisticated calibration techniques like Approximate Bayesian computation and Monte Carlo Calibration. For this purpose the validation of model parameters is warranted with sufficient number of patient including long term data such as re-treatment of the same site. Although the model applied to 67NR tumor experiment in this study cannot be validated beyond 30 d after treatment due to the lack of measurement data, the extrapolation may be still useful to provide important insight on radio-immune response of the body. The insight gained from the study of model would allow to efficiently design clinical studies aiming to amplify the synergistic effect of the radiation and immune therapies.

In our study, the mathematical model was applied to each individual voxel in the tumor in order to consider the effect of heterogeneous dose distribution. Tumor cells in each voxel went through different dynamics due to the dose difference. The immune effect, *Z* was assumed to be effectively diffused between the voxels and therefore the average value of immune effect is applied to all tumor cells. The nature and range of bystander signalling is complex phenomenon as a function of diffusing molecular factors. Optimal diffusion in human cancer is yet to be discovered. Peng *et al* (2018) investigated three different diffusion models (short, medium and long range) for bystander effect. Their model outperformed the classical LQ model to fit the observed survival in both uniform and modulated radiation fields. Among three models, long range diffusion model using average of the dose gradient gave the best overall prediction. Taking average value of immune response is similar idea of the work from Peng *et al* (2018) but needs validation in future work.

## 5. Conclusion

In this study we present a mathematical model of tumor growth considering the immune response of the body and the counterpart immune suppression effect of the tumor during radiation therapy. Two distinctive modes of tumor status were identified and analyzed: immune limited and immune escape. Tumors in the immune limited mode can grow only up to a finite size and the tumor volume (terminal tumor volume, *T*_∞_) in this equilibrium can be analytically found. In the immune escape mode, there is a critical tumor mass for successful radiotherapy and it would be beneficial to start treatment early with enough dose before tumors grow more than the critical mass. The mechanism of SFRT was explained well by the development of immune response using the proposed mathematical model. Although the elevated immune response from SFRT often compensates less radiation damage in unirradiated region and produces longer tumor control effect, special attention should be paid when SFRT is considered especially tumor volume is approaching to the critical tumor volume. Therefore, deeper understanding of immune process would be a key for the successful application of the new cancer treatment. This study may provide some insight on the role of immune response for cancer patients by the analysis on the tumor model.

## Data Availability

All data that support the findings of this study are included within the article (and any supplementary information files).

## Conflict of interest

The authors have no conflict of interest to report.
